# A coral-on-a-chip microfluidic platform enabling live-imaging microscopy of reef-building corals

**DOI:** 10.1038/ncomms10860

**Published:** 2016-03-04

**Authors:** Orr H. Shapiro, Esti Kramarsky-Winter, Assaf R. Gavish, Roman Stocker, Assaf Vardi

**Affiliations:** 1 Department of Plant and Environmental Sciences, Weizmann Institute of Science, Rehovot 76100, Israel; 2 Department of Biotechnology Engineering, Ben-Gurion University of the Negev, Beer-Sheva 84105, Israel; 3 Department of Civil and Environmental Engineering, Massachusetts Institute of Technology, Cambridge, Massachusetts 02139, USA; 4 Department of Civil, Environmental and Geomatic Engineering, ETH Zurich, Zurich 8093, Switzerland

## Abstract

Coral reefs, and the unique ecosystems they support, are facing severe threats by human activities and climate change. Our understanding of these threats is hampered by the lack of robust approaches for studying the micro-scale interactions between corals and their environment. Here we present an experimental platform, coral-on-a-chip, combining micropropagation and microfluidics to allow direct microscopic study of live coral polyps. The small and transparent coral micropropagates are ideally suited for live-imaging microscopy, while the microfluidic platform facilitates long-term visualization under controlled environmental conditions. We demonstrate the usefulness of this approach by imaging coral micropropagates at previously unattainable spatio-temporal resolutions, providing new insights into several micro-scale processes including coral calcification, coral–pathogen interaction and the loss of algal symbionts (coral bleaching). Coral-on-a-chip thus provides a powerful method for studying coral physiology *in vivo* at the micro-scale, opening new vistas in coral biology.

## Abstract (web summary)

Studies of coral in laboratory settings are limited by a lack of reliable methods for manipulation of the coral microenvironment and monitoring of coral processes *in vitro*. Here the authors develop coral-on-a-chip, a microfluidic platform to enable study of coral processes at single-cell resolution.

## Graphical abstract

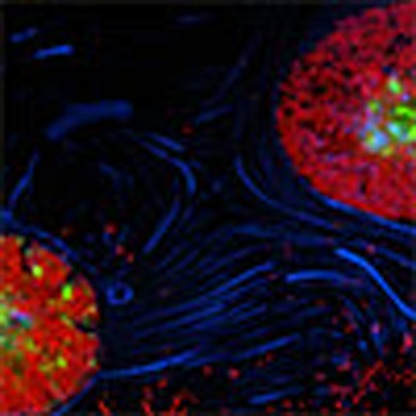

Reef-building corals (order Scleractinia) harbour a complex network of interactions between the coral animal, photosynthetic algal symbionts and a complex bacterial community^1^. This partnership allows corals to proliferate in tropical, nutrient-poor waters, where they form the backbone of the coral-reef ecosystem and its associated biodiversity^2^. Further, while covering less than 0.1% of the ocean surface, the activity of coral reefs has a significant impact on global climate, as coral calcification is estimated to sequester 70–90 million tons of carbon per year^3^, exceeding 10% of marine calcium carbonate mineralization. Over the past several decades, concomitant with global rise in sea-surface temperatures, coral reefs have undergone continual decline due to disease outbreaks, including mass bleaching events^4^,^5^, leading to a large increase in research efforts into the physical and biological processes governing coral-reef health and proliferation under changing environmental conditions^3^,^6^,^7^,^8^,^9^.

Reef corals typically form structures on the order of centimetres to metres, yet the majority of biological activity takes place at a thin layer of living tissue, 0.1–5 mm depending on species, enveloping the coral skeleton^3^,^10^. Studying corals at the colony scale therefore fails to capture biological processes occurring at the tissue or cellular levels, and how these processes are affected by environmental stress. Tissue or cellular level coral physiology has been studied extensively using microscopic methods including histology, electron microscopy and more recently nanoscale secondary ion mass spectrometry^11^,^12^,^13^,^14^. While these methods provide a wealth of information regarding cellular and subcellular structures, they require fixation of tissues prior to imaging and so are limited in their ability to follow dynamic processes *in vivo* at relevant time scales. An alternative approach is the generation of miniaturized model systems in the form of cell or tissue cultures that could be studied *in vitro* in real time. Such miniaturized model systems have yielded valuable insights into the physiological processes involved in symbiosis and calcification^15^,^16^,^17^,^18^,^19^. However, the process of disrupting the coral tissue to generate cell or tissue cultures necessarily results in loss of the tissue organization and hence the organismal context of resulting observations. Moreover, as all coral cell or tissue cultures reported to date have limited lifespans of up to 3 weeks^15^,^16^,^18^, the cells or tissues in such cultures are in a state of declining health, likely stressed and thus may not represent the full spectrum of coral physiological and metabolic processes.

Model system miniaturization can be achieved through micropropagation, the production of tissue clones that ultimately develop into whole organisms^20^,^21^. Unlike cell or tissue cultures, micropropagation preserves much of the organization of the whole organism, enabling the study of cells or tissues in their uncompromised physiological state. Furthermore, micropropagation enables the rapid production of a large number of clonal organisms with highly similar life histories, thus reducing complexity and simplifying experimental design. Such systems have been particularly useful in the plant sciences, where they are routinely used for comparative analysis of tissues or whole organisms, plant crop production, and conservation and restoration^20^,^22^,^23^,^24^,^25^. A number of, micropropagation systems have been developed for cnidarian species, most notably the freshwater *Hydra* polyp^26^,^27^, the sea anemones *Aiptasia* and *Nematostella*^1^,^28^,^29^ and more recently the solitary scleractinian coral *Fungia granulosa*^21^,^30^.

Combining micropropagates with recently developed microfluidic techniques offers a particularly powerful tool for studying live organisms at the tissue or cellular level. The development of microfluidic flow systems, where environmental parameters can be precisely controlled and monitored, together with advances in microscopic imaging techniques have opened new frontiers in the study of cellular and microbial systems^31^,^32^,^33^, organs^34^, and whole organisms^35^. Recently, we demonstrated the use of microfluidics in studying the chemotactic response of a bacterial pathogen towards coral mucus^36^, as well as the dynamic microenvironment generated by ciliary vortices at the surface of reef-building corals^37^. Introducing coral micropropagates into a microfluidic setting has the potential to provide a powerful new platform for *in vivo* visualization of coral physiological processes at microscopic resolution, under controlled conditions and over extended time periods. To this end, we developed a robust method for the micropropagation of the reef-building coral *Pocillopora damicornis*, and two additional coral species, and for the integration of resulting micropropagates into a microfluidic flow system. We demonstrate the strength of this novel system, which we term coral-on-a-chip, by performing live-imaging microscopy of eco-physiological processes in reef-building corals, providing a new and powerful experimental approach to study the biology and ecology of reef corals.

## Results

### Micropropagation of coral polyps

**Figure 1:**
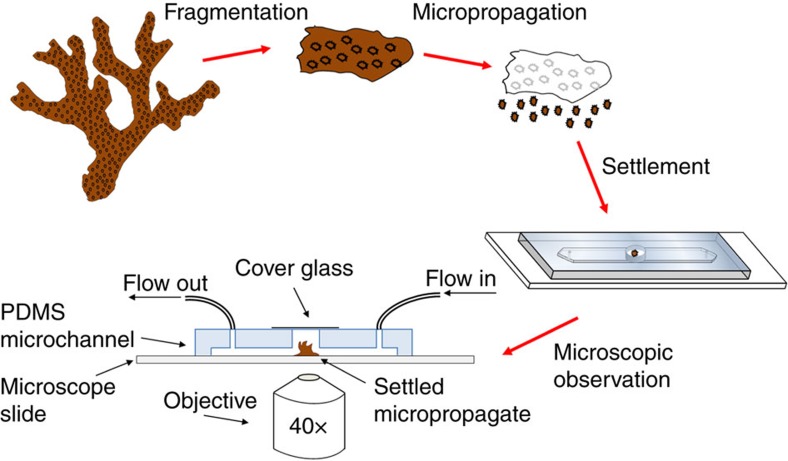
Schematic illustration of the workflow for setting up the coral-on-a-chip platform. A branch tip fragmented from a healthy coral colony is micropropagated via a polyp-bail-out response by subjecting it to a gradual increase in salinity. Micropropagates are transferred to open microwells within a microfluidic channel and incubated under controlled light, flow and temperature conditions to induce settlement. Microscopic observation is facilitated by sealing the microwell with a glass coverslip and introduction of flow via silicon tubes connected to a syringe pump. Light intensity is controlled using the microscope's transmitted illumination system. Temperature control may be provided using a stage incubator. The coral-on-a-chip platform allows maintaining live coral micropropagates on the microscope stage for extended time periods while precisely controlling the chemical and physical environment.

**Figure 2:**
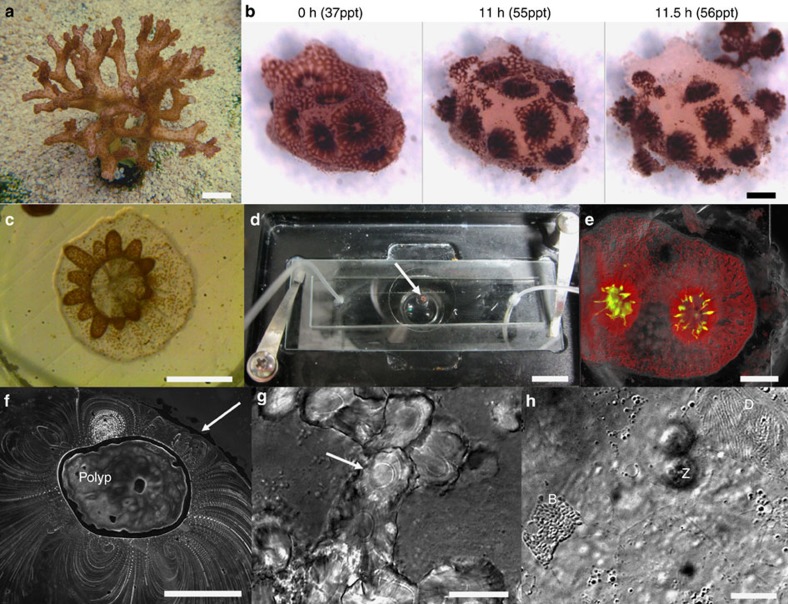
Explantation and resettlement of *P. damicornis* micropropagates into the coral-on-a-chip microfluidic platform. (**a**) A colony of *P. damicornis.* Polyps appear as brown dots covering the colony surface. (**b**) Under salinity stress, polyps from a small *P. damicornis* branch (0 h) are separated from each other through rupturing of the coenosarc tissue (11 h). Polyps are then ejected from the skeleton (11.5 h) resulting in the production of clonal micropropagates. (**c**) A settled *P. damicornis* micropropagate, 48 h following explantation. The polyp base is flattened against the glass surface. Extended tentacles (brown) indicate recovery from the stress experienced during the explantation process. Symbiotic algae (zooxanthellae) are visible as brown dots. (**d**) A coral micropropagate (white arrow) settled inside the coral-on-a-chip microfluidic device, held on a temperature-controlled microscope stage. Tubes attached to the microfluidic device allow the introduction of flow and the control of water chemistry during the experiment. (**e**) A *P. damicornis* micropropagate as seen by epi-fluorescence microscopy, based on the autofluorescence of the coral's native GFP (green) and algal chlorophyll (red) (See Supplementary Fig. 1 for additional coral species). (**f**) Vortical ciliary flows around a settled *P. damicornis* micropropagate, visualized from the motion of small tracer particles suspended in the water (please see the study by Shapiro *et al*.^37^ for methodology). The white arrow points to the edge of the microwell. (**g**) Calcium carbonate crystals deposited by a *P. damicornis* micropropagate, showing concentric growth rings (white arrow) possibly reflecting diel growth cycles. (**h**) High-magnification view of the calicoblastic tissue immediately above the glass substrate, demonstrating the ability to track key components of the coral holobiont. Cell borders are seen as faint straight lines. D—desmocyte anchoring the coral to the glass surface. Z—symbiotic zooxanthellae algae in the coral's gastrodermis, slightly above the focal plane. B—a bacterial micro-colony growing between the glass surface and the coral's calicodermis. (See Supplementary Fig. 3 for full image) Scale bars: (**a**,**d**) 1 cm; (**b**,**c**,**e**,**f**) 500 μm; (**g**,**h**) 20 μm.

A schematic representation of the coral-on-a-chip system is shown in Fig. 1. Coral micropropagates are generated by polyp explantation induced by a natural stress response previously termed ‘polyp-bail-out'^38^. We induce this type of response in a controlled manner by subjecting coral fragments to a gradual increase in salinity, to which *P. damicronis* corals (Fig. 2a) used in our experiments respond in a highly predictable manner (Fig. 2b; Supplementary Movie 1). Polyp retraction is typically observed at salinities of 42–45 p.p.t., followed by stretching and thinning of the coenosarc (the tissue connecting adjacent polyps) at ∼50 p.p.t. Severing of the coenosarc occurs at 52–54 p.p.t., rapidly followed by separation of the polyp tissue from the skeleton. It should be noted that the precise salinity for each stage of the explantation process varies, depending on colony health and possibly also on its origin. Nevertheless the occurrence of polyp-bail-out on salinity increase and the sequence of steps described above are robust and lead to the expulsion of over 90% of the polyps from each fragment.

Using a similar approach, we induce polyp explantation in two additional coral species within the family Pocilloporidae (Supplementary Fig. 1). Explantation in the bird-nest coral *Seriatopora hystrix* is induced through a more gradual increase in salinity compared with *P. damicronis*, going from 39 to 52 p.p.t. over a period of 44–48 h. In *Stylophora pistillata*, stretching and thinning of the coenosarc tissue is induced in a similar manner to *P. damicornis*, but is not followed by polyp expulsion. This final step is induced by exposing of the salinity-stressed coral (52–54 p.p.t.) to air for a period of 30 min before continuing incubation under the same high-salinity conditions.

### Resettlement of micropropagated coral polyps

**Figure 3:**
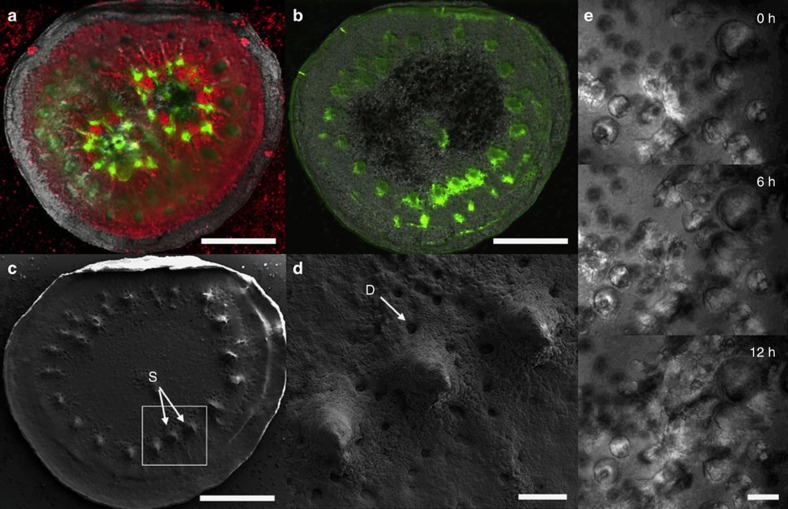
The coral-on-a-chip platform as a model system for studying coral calcification. Coral micropropagates resume calcification soon after resettlement and proceed to secrete a skeleton that has many of the features of the mother colony. (**a**) A micropropagate consisting of two *P. damicornis* polyps, 6 weeks following resettlement. Coral native GFP (green) and chlorophyll autofluorescence (red) are shown on the background of the newly formed skeleton (imaged using polarized light microscopy). (**b**) Skeleton of the same micropropagate from **a** following calcein labelling and tissue removal. Calcein (green) is preferentially incorporated at the edge of the tissue or at sites corresponding to tissue areas devoid of algal symbionts. (**c**) Skeleton from **b** imaged with scanning electron microscopy (SEM). Calcein labelling (green areas in **b**) is strongest at skeleton spinules (S) and at the calyx edge. (**d**) Higher magnification view of the area marked by the white rectangle in **c** showing skeletal spinules (S) that are strongly labelled with calcein in **b**. Desmocyte scars (D) mark the locations of cells anchoring the polyp tissue to the calcium carbonate skeleton. (**e**) High-magnification view (× 40 objective) of the formation of aragonite crystals by a newly settled coral micropropagate (48 h from explantation) held in a microfluidic channel with continuous flow of FASW, visualized using differential interference contrast microscopy. (See also Supplementary Movie 3). Scale bars: (**a**–**c**) 200 μm; (**d**) 50 μm; (**e**) 20 μm.

Explanted polyps from all three coral species can be induced to settle onto glass surfaces such as petri dishes, microscope slides or coverslips. Gross polyp morphology, including polyp mouth (stomodaeum) and tentacles, is retained throughout the micropropagation and settlement process. To induce settlement, micropropagates are placed in small microwells (3 mm diameter × 1 mm depth) fabricated on a microscope slide. Slides with micropropagates are incubated in a custom raceway system under controlled light, temperature and flow conditions (Supplementary Fig. 2). Initial attachment to the glass surface is observed within 12 h of polyp explantation, followed by flattening of the polyp base against the glass surface and extension of tentacles over a period of 24–48 h (Fig. 2c). Calcification (Figs 2g; 3) is observed within 2–4 days of settlement. Settled micropropagates can be maintained for periods of weeks to months, either in the raceway system or in aquaria. Under the experimental conditions used here, up to 90% of micropropagates from a single *P. damicornis* fragment could be induced to settle onto glass microscope slides. However, this rate of success depends on colony health and water quality, with settlement rates in some of our experiments dropping below 10% when conditions in the aquarium deteriorated.

### The coral-on-a-chip microfluidic system

The small size and transparency of the micropropagates make them ideally suited for live-imaging microscopy. Epifluorescence microscopy of settled micropropagates is facilitated by the strong autofluorescence of both the coral's native green fluorescent protein (GFP) and the algal chlorophyll, without the need for fluorescent probes (Fig. 2e; Supplementary Fig. 1). The use of transmitted light microscopy reveals additional micro-scale features of the coral micropropagates such as vortical flows driven by the coral's epidermal cilia^37^ (Fig. 2f). High-magnification microscopy (× 60 or higher) if facilitated by settling coral micropropagates on glass coverslips. The use of differential interference contrast (DIC) allows the observation of fine details such as the daily growth rings within newly deposited skeletal features (Fig. 2g), as well as coral cell boundaries or bacterial microcolonies forming in or around the coral polyp (Fig. 2h; Supplementary Fig. 3).

To enable long-term microscopic observation under controlled environmental conditions, we settle micropropagates inside a simple microfluidic device consisting of a shallow channel etched in polydimethylsiloxane (PDMS), with inlet and outlet ports and an open microwell (Figs 1; 2d). Micropropagates are induced to settle in the microwell as described above. Prolonged microscopic observation under constant conditions is facilitated by reversibly sealing the microwell and the introduction of flow via the inlet and outlet ports. This prevents unwanted changes in the coral's microenvironment due to evaporation or accumulation of waste materials during the observation period. Maintaining a constant flow further enables the precise and continuous control over water chemistry in the microchannel, as well as the introduction of biological or chemical agents as part of the experimental design.

### *In vivo* imaging of calcification and skeletogenesis

Coral calcification and skeletogenesis forms the backbone of the coral-reef ecosystem. By adding the fluorescent dye calcein, which is incorporated into growing calcium carbonate crystals^39^,^40^, to the channel inflow, we were able to visualize patterns of calcium carbonate deposition within a settled coral micropropagate (Fig. 3a,b). Calcein incorporation was subsequently correlated to skeletal morphology imaged using scanning electron microscopy (SEM) (Fig. 3c,d). Using this approach, we demonstrate the preferential incorporation of calcein by a settled miropropagate into protruding skeletal elements resembling the septa and spinules found in mature coral skeletons.

Using the coral-on-a-chip platform, we can follow the growth of specific crystal bundles using time lapse microscopy at high magnification over periods of hours to days. We present results from a 12-h experiment, where skeleton deposition by a newly settled *S. pistillata* micropropagate is imaged every 10 min at × 40 magnification using DIC microscopy (Fig. 3e; Supplementary Movie 2). We observed intermittent crystal growth, with periods of rapid elongation (>2 μm h^−1^) interspersed by periods of no measurable growth. Growth is highly heterogeneous, with crystals separated by as little as 10 μm showing substantially different growth rates and patterns.

### Micro-scale study of coral bleaching and disease

**Figure 4:**
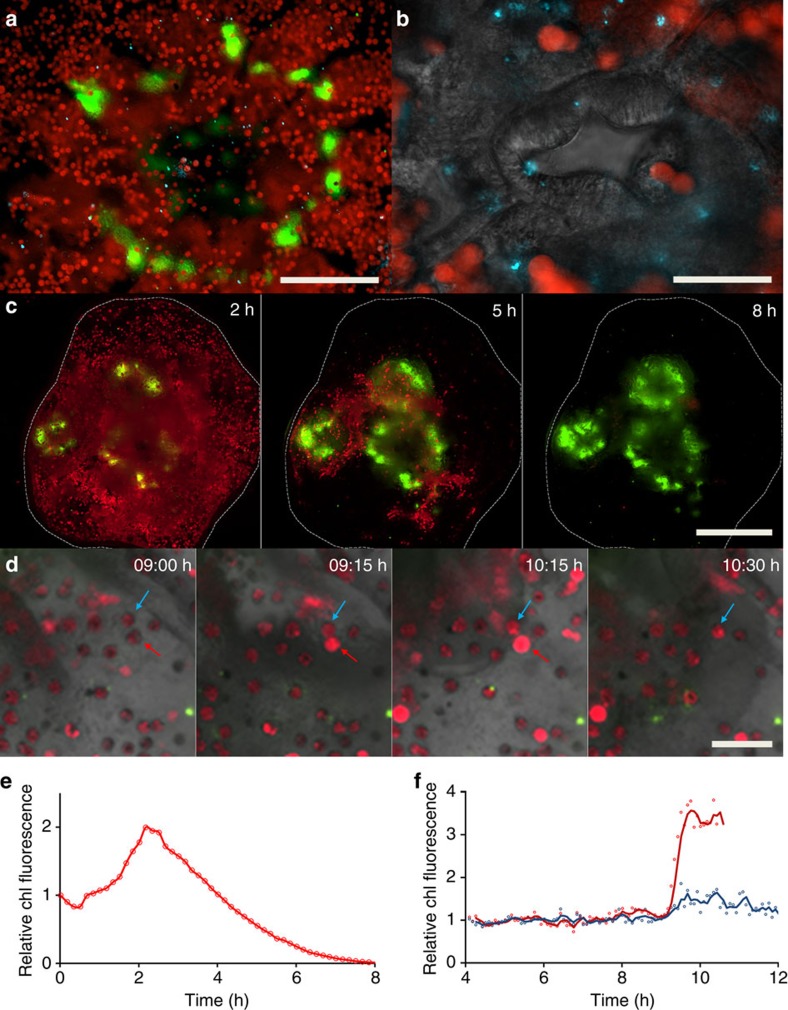
Using the coral-on-a-chip platform for the visualization of bacterial infection and coral bleaching. A detailed view of microbial processes and interactions within the coral holobiont. (**a**) The aboral view provided by the coral-on-a-chip platform enables the visualization of fluorescently tagged bacterial cells inside the coral's gastrovascular cavity. Zooxanthellae (red) and DsRed-tagged bacterial pathogens (blue dots) are seen on the background of the coral's native GFP (green). (**b**) Higher magnification view of the mouth area. Algal cells and bacterial pathogens are seen on the background of the polyp's mouth, here visualized by differential interference contrast microscopy. (**c**) Bleaching is induced in a micropropagate subjected to high light intensity (2,500 μmol photons per m^2^ per s), seen through the gradual decrease in the autofluorescence signal from the algal chlorophyll. Individual polyps are seen by their native GFP fluorescence, concentrated around the mouth and tentacles. (**d**) Part of a bleaching process similar to **a** but under reduced light intensity (1,500 μmol photons per m^2^ per s). Algal chlorophyll and native GFP are overlaid on a bright field image of the coral tissue. This image sequence compares two individual algal cells that appear to be physically linked as one (indicated by red arrow) is lost while the other (blue arrow) remains. (**e**,**f**) Changes as a function of time of total chlorophyll (chl) fluorescence of whole micropropagate (**c**) and algal cells pointed by arrows (**d**). Values in **e** denote normalized chlorophyll fluorescence for the area marked by a white dashed line, corresponding to the settled micropropagate in **c**. Values in **f** denote total chlorophyll fluorescence per marked alga in **d**, divided by the average fluorescence for that alga from 4 to 8 h of the experiment. Red and blue curves denote algal cells pointed by red and blue arrows, respectively. Scale bars: (**a**) 200 μm; (**b**) 50 μm; (**c**) 500 μm; (**d**) 50 μm.

The coral-on-a-chip platform allows us to follow bleaching and disease processes in live coral polyps at the single-cell level (Fig. 4). Using our system, we can introduce fluorescently tagged pathogens into the microchannel, and to follow their interaction with the coral micropropagate by live-imaging microscopy. To overcome the autofluorescent background of the coral host, we transformed the established coral pathogen *Vibrio coralliilyticus* strain YB2 (ref. 41) with a plasmid encoding for the DsRed.T3 fluorescent protein (Fig. 4a,b). The emission wavelength of this protein, together with its high quantum yield, makes it readily distinguishable from the strong autofluorescence of both the coral's native GFP and the chlorophyll of its algal symbionts. By adding these transformed pathogens to the microfluidic channel we were able to follow the infection process in real time, including the accumulation *V. coralliilyticus* cells within the polyp's gastrovascular cavity (Fig. 4a,b, blue dots).

By exposing settled micropropagates to high light intensities we can induce the coral to lose its algal symbionts, thus bringing the process of coral bleaching into our microfluidic channel. By this method, we can observe and measure the bleaching process at high spatio-temporal resolution. This is demonstrated here for a micropropagate consisting of three coral polyps, where exposure to high light intensity (2,500 μmol photons per m^2^ per s) over a period of 8 h results in the near-complete loss of its zooxanthellae (Fig. 4c; Supplementary Movie 3). Bleaching is not uniform, but appears to progress from the coenosarc tissue towards the polyps' centre. Using simple image analysis we extracted quantitative measurements of chlorophyll autofluorescence for the entire micropropagate, revealing a doubling in fluorescence intensity (Fig. 4e). These results were further validated by studying the bleaching process at single-cell resolution, providing additional insights into the micro-scale dynamics of coral bleaching. Specifically, our results demonstrate different response kinetics for neighbouring individual symbionts (Fig. 4d,f). One striking result is that the loss of an algal cell is often preceded by elevated fluorescence intensity of its chlorophyll. In the example given here, the chlorophyll autofluorescence of the alga marked by a red arrow (red curve in 4f) is first seen to intensify at 0915 hours. It then remains high for the next hour, after which the algal cell disappears. A neighbouring symbiont, which appears to be physically linked to the affected cell (blue arrow; blue curve in 4f), is relatively unaffected during the capture period.

## Discussion

The coral-on-a-chip microfluidic platform presented here allows direct microscopic observation and measurement of fundamental behaviours and physiological phenomena in live corals at previously unattainable spatio-temporal resolutions. The ability to tightly control the physical and chemical parameters within the microfluidic chamber further provides us with the ability to study coral responses to various external effectors and environmental insults at the micro-scale.

The use of micropropagation preserves much of the complexity of both the coral holobiont and its microenvironment. Coral micropropagates are generated through a natural stress response described previously as ‘polyp-bail-out'^38^. When exposed to sub-lethal stress, coral polyps are expelled from the mother colony, possibly as a means to escape adverse local conditions^38^,^42^,^43^. Micropropagated polyps remain viable and have the potential to settle onto a solid surface to form microcolonies. Here we demonstrate the applicability of this approach to three coral species. We expect further efforts to add additional species to this list, facilitating the establishment of one or several coral model systems.

Our work describes the first use of micropropagated coral polyps as a micro-scale experimental platform. The scale of observation, the minute amount of tissue and the small working volume differ substantially from conventional experimental macro-scale approaches in coral studies, where the unit of observation is usually a small colony or branch, either *in situ* or *in aquaria*. This system also differs from the use of dissociated coral cells or cell aggregates, previously suggested as a possible alternative for micro-scale studies of coral physiology^15^,^16^,^17^. Cell dissociation inevitably results in loss of tissue spatial organization, altering the physical and chemical microenvironment experienced by the studied sample. The unique microscopic view of settled coral micropropagates thus opens a new window into coral physiology at resolutions down to the single-cell level. The clonality of multiple micropropagates derived from a single mother colony simplifies experimental design by reducing genetic variability, allowing to reliably compare the effects of different treatments. The ability to integrate coral micropropagates into a microfluidic device allows, for the first time in coral studies, the use of ong-term microscopic observation, and opens the use of a whole suite of analytical tools developed for similar microfluidic platforms^44^,^45^.

To demonstrate the applicability of our coral-on-a-chip system, we focus on three questions that are currently at the heart of coral-reef research, that is, calcification, the breakdown of symbiosis under environmental stress (coral bleaching) and coral disease. Calcification and skeletogenesis, processes that underlie the development of coral reefs^17^,^46^,^47^, are among the most studied aspects of coral physiology. These subjects have received increased attention in light of global environmental changes, particularly ocean acidification^43^,^48^,^49^. Nevertheless, the underlying micro-scale processes of calcification remain poorly understood, largely because of the difficulty in observing the calcifying layer in the live coral without disturbing the calcification process^47^,^50^,^51^. In light of the importance of the calcification process, and the research attention it receives, it was imperative to first verify that calcification in coral micropropagates is representative of that in intact colonies. To do so, we used the fluorescent dye calcein, which is incorporated into calcium carbonate crystals^39^,^40^ during skeletal growth (Fig. 3a–d). Calcein is known to be preferentially incorporated at specific sites within the coral skeleton, designated centres of calcification^47^,^50^, typically associated with protruding skeletal elements such as spinules and septa. Our results indeed demonstrate localized calcein incorporation (Fig. 3b) at protruding elements subsequently shown to resemble spinules and septa by SEM imaging (Fig. 3c,d). These results indicate that coral micropropagates indeed retain the basic calcification and skeleton morphology properties of the mother colony, and are thus a promising experimental system for the microscopic study of these important processes.

The high spatial resolution afforded by the coral-on-a-chip platform enables the use of polarized or DIC microscopy for real-time visualization of *de novo* calcification. This allows us to record and quantify the deposition of aragonite crystals under highly controlled environmental conditions. We demonstrate this ability using the coral *S. pistillata* (Fig. 3e; Supplementary Movie 2), which is among the most used model species for the study of coral calcification^16^,^40^,^52^. The ability to precisely control and manipulate the environmental parameters experienced by the coral micropropagate will enable future studies into the effects of different environmental stresses on the deposition of specific skeletal elements. Only one system to date, the lateral skeleton preparative (LSP) assay^52^, provides comparable spatial resolution when studying *in vivo* coral calcification. Using the LSP essay, the expanding edge of a small coral fragment attached to a glass slide can be imaged microscopically. Our system offers several advantages over LSP preparations, including improved clonality, shortened generation times (from several weeks to just a few days), and most importantly, flexible experimental design due to the ability to precisely control the environmental parameters during microscopic imaging. Thus, coral-on-a-chip provides a unique platform for studying the micro-scale processes underlying coral calcification.

Coral-on-a-chip provides an additional advantage in that it allows the long-term observation of specific components of the coral holobiont during bacterial infection and coral bleaching. Coral disease is increasingly threatening coral reefs, exacerbated by a rise in both global seawater temperatures and anthropogenic disturbances^8^,^53^,^54^. Here we demonstrate the use of the coral-on-a-chip platform to study the infection of a *P. damicornis* micropropagate with its well-established pathogen, *V. coralliilyticus*^41^,^55^, a major cause of coral disease. By fluorescently tagging the bacterial pathogens, we were able to show how, during the infection process, *V. coralliilyticus* enters the gastrovascular cavity via the polyp's stomadeum (Fig. 4a,b). This result suggests a hitherto under-explored route to coral infection, while representing a first *in vivo* observation of a bacterial pathogen inside a coral polyp.

To induce coral bleaching we exposed micropropagates settled within our coral-on-a-chip system to high light levels. This resulted in a rapid and repeatable breakdown of the coral–algae symbiosis, which allowed us to track and measure the loss of algal symbionts in real time at both the polyp and single-cell levels (Fig. 4c–f; Supplementary Movies 3 and 4). We observed a sharp increase in the autofluorescence of algal chlorophyll as part of the bleaching process, suggesting breakdown of the photosynthetic process^56^. Our results indicate that susceptibility of the algal symbionts to high light conditions is affected by their localization within the coral polyp, and possibly by some degree of innate variability. Moreover, the loss of symbionts is shown to be highly selective, with cells separated by only a few micrometres showing different bleaching kinetics. This suggests specific signalling between coral and algae, possibly mediated by the induction of reactive oxygen species^57^,^58^,^59^, which needs to be further explored.

The rapid generation of multiple, clonal coral micropropagates, combined with a microfluidic platform for their long-term microscopic study, provides a substantial advancement for coral studies. The coral-on-a-chip platform brings the power of animal-on-a-chip systems, along with their unique range of microscopic, biochemical and analytical tools^35^,^58^,^59^, to the study of coral biology. Our microfluidic device may also be used to study newly settled coral larvae, providing an important tool for comparing coral physiological processes during different life stages^53^. Combined with recent advances in coral genomics^60^,^61^, together with emerging genome editing tools such as short interfering RNA^62^ and the CRISPR–CAS9 system^63^,^64^, our system will provide an important and powerful platform for the introduction of forward and reverse genetics into the study of reef-building corals. As corals represent an early stage in the evolution of multicellular organisms, the coral-on-a-chip platform has the potential to establish them as an important model system for studying the evolution of complex traits such as tissue organization, symbiosis and biomineralization.

## Methods

### Coral growth conditions

Small colonies of the coral *P. damicornis* collected by permit (permit no# 2014/40327) from the reef opposite the Inter-University Institute in Eilat, Israel were kept in a 200-l aquarium equipped with a biological filtration system under the following conditions: Temperature: 24 °C; Salinity: 39 p.p.t. (artificial seawater prepared by dissolving Reef Salts (Aqua Medic GMBH, Germany) in deionized water); light intensity: 200 μmol photons per m^2^ per s, 12-h light–dark cycle. Light was provided using an equal proportion of white and actinic blue T5 fluorescent tubes.

### Coral micropropagation

For each experiment, a small (∼5 mm) branch tip is removed from the mother colony using a clean stainless-steel bone cutter. The branch tip is placed in an open glass Petri dish filled with filtered artificial seawater (FASW) just covering the coral fragment. A gradual increase in salinity due to water evaporation results in a polyp-bail-out response, with separate polyps released from the coral skeleton. Polyp release can be expedited by gently pipetting or stirring the water around the coral fragment. Precise conditions for the different coral species used here differed as detailed in the text. A 5-mm fragment from the corals *P. damicornis* or *S pistillata* typically yields 30–40 micropropagates. Expelled micropropagates are transferred to a small Petri dish containing FASW at ambient salinity for up to 10 min for initial recovery. Micropropagates are then screened for viability using a stereo-microscope, and scored by observing tissue integrity, the extension of mesenterial filaments and the presence of vortical ciliary flows^37^. Viable polyps are collected using a clean glass pipette and used for settlement as described. Polyp-bail-out imaging (Fig. 2b; Supplementary Movie 1) was done using an Olympus SZX12 stereoscope (Olympus, Japan) equipped with a UI-1465LE camera (IDS Imaging Development Systems GmbH, Obersulm, Germany).

### Microfluidic channels

A simple straight channel (4 cm × 3 mm × 0.1 mm) is etched into a silicone elastomer (PDMS); (Sylgard 184, Dow Corning) using soft lithography^35^,^36^. Inlet and outlet holes are punched at both ends of the channel using a 1-mm biopsy punch (AcuDerm, FL, USA), and a 3-mm punch is used to make a well at the centre of the channel. Channel is placed on the clean surface of a new glass microscope slide or a 60 × 24 mm cover slip (Sail brand, China) without plasma bonding.

### Raceway flow system

The raceway is made of a shallow glass tray divided into three channels, each 3-cm wide and 1.5-cm deep, with a 1-cm-high downstream wall to direct overflow. All glass parts are glued together using non-toxic silicone aquarium sealant. To maintain flow, the raceway is placed on top of a small glass container (20 × 30 cm area; 10-cm deep) filled with FASW. Glass brackets placed about 3 cm below the top of the container support a 19 × 29 cm glass shelf, so that a small gap is left between the edge of the shelf and the container's short sides. The total operating volume is ∼3.5 l. A 20 W submersible aquarium pump (Aqua Medic GMBH) placed inside the glass container drives constant flow through a silicone tube coming out of the gap on one end of the glass shelf and divided between the raceway channels. The system is placed at a slight angle so that water coming out of the raceway channels return to the main container through the gap on the downstream end, thus allowing continuous recycling of the water. Water temperature is maintained using a self-regulated 25 W submersible aquarium heater (Aqua Medic) and an aquarium thermometer is used to visually verify temperature. Salinity is controlled by daily addition of distilled water to compensate for evaporation. Light is provided using a mixture of white and blue LED strips, with a total photon flux of 150 μmol photons per m^2^ per s (measured using a Li-Cor 189 light meter (Li-Cor, USA)) and operated on a 12-h light-dark cycle.

### *V. coralliilyticus* transformation and growth conditions

A tri-parental conjugation protocol was used to transform *V. coralliilyticus* strain YB2 by a plasmid carrying a gene encoding for a DsRed2 fluorescent protein [DsRed.T3(DNT),^65^]. Following transformation, colonies formed on marine agar (Marine Agar 2216, Difco, USA) supplemented with 50 mg l^−1^ Kanamycin (Sigma) were screened for DsRed fluorescence. Transformed *V. coralliilyticus* colonies were regrown in Marine Broth 2216 and in −80 °C in 15% glycerol. For infection experiments, DsRed-tagged *V. coralliilyticus* were grown overnight in 1/1,000 marine broth diluted in FASW containing 50 mg l^−1^ Kanamycin. Cells were washed in FASW prior to their introduction into the microfluidic channel.

### Experimental set-up and light microscopy

The explant-containing channel is mounted on a motorized XY stage (Prior Scientific, MA, USA) with a temperature-controlled inset (LCI, Korea). The microwell is sealed by gently scraping the top surface of the microchannel and placing a clean cover glass on top of the open well. Care is taken to avoid trapping of bubbles that may interfere with transmitted light microscopy. The inlet tube (Fig. 2d) connects one end of the channel to a reservoir containing the incoming water. The outlet tube from the other end of the channel is connected to a syringe pump (New Era Pump Systems, NY, USA) set to withdraw mode, ensuring negative pressure in the channel to minimize leakage. For all experiments reported here, flow rate was set to 1 ml h^−1^. Light microscopy imaging was performed using an Olympus IX81 microscope (Olympus) equipped with × 4 (numerical aperture (NA) 0.13), × 10 (NA 0.3), long working distance × 40 (NA 0.6) and a × 60 oil (NA 1.42) objectives, and a motorized stage (Prior Scientific). A Lumen 200PRO illumination system (Prior Scientific), enabling rapid switching of excitation and emission wavelengths, was used for epi-fluorescence microscopy. Images were captured using a Coolsnap HQ^2^ CCD camera (Photometrics, Tuscon, AZ, USA) and processed using the CellSens Dimension V1.11 software package (Olympus). Light flux for all experiments was provided using the microscope's transmitted light, and measured using a Li-Cor 189 light meter.

### Calcein staining

A microfluidic device holding a coral micropropagate was placed on the microscope stage. Illumination was set to 500 μmol photons per m^2^ per s. Calcein was introduced into the chamber for a period of 1 h by filling the inlet reservoir with a 20 μM calcein solution in FASW. After 1 h, inlet was changed to FASW and free calcein washed out of the channel. Inlet was then change to 1% sodium hypochlorite in FASW for 1 h to remove tissues and organic residues, as well as unincorporated calcein. Filters used for epi-fluorescence microscopy were: GFP and Calcein—Ex:490 nm/Em:535/50 nm; Chlorophyll—Ex:490 nm/Em: 660/50 nm. All filters were obtained from Chroma (Chroma Technology Corp, Bellows Falls, VT USA). SEM was performed by removing the PDMS channel and cutting the glass slide around the exposed skeleton using a hand-held glass cutter. Skeleton was dehydrated by immersion in 100% ethanol, dried under vacuum and sputter-coated with gold–palladium (Edwards, S150). Coated skeleton was imaged using a Carl Zeiss Ultra 55 scanning electron microscope (Carl Zeiss, Oberkochen, Germany).

### Bacterial infection

A suspension of ∼10^8^ cells per ml of DsRed-labelled *V. coralliilyticus* was introduced into the channel for a period of 2 h. Inlet was then changed to FASW and micropropagate held on the microscope stage for an additional 7-h period. Micropropagate was imaged throughout the process at 15-min intervals. Filters used for epi-fluorescence microscopy were: GFP—Ex:490 nm/Em:535/50 nm; Chlorophyll—Ex:490 nm/Em: 660/50 nm; DsRed—Ex:555/20 nm/Em: 590/33 nm.

### Coral bleaching imaging and quantification

To induce bleaching, that is, loss of algal symbionts, micropropagates were incubated for 8 h under 2,500 μmol photons per m^2^ per s (Fig. 4c; Supplementary Movie 3) or 12 h under 1,500 μmol photons per m^2^ per s (Fig. 4d; Supplementary Movie 4). Images were captured at 5- or 10-min intervals as noted, with each frame composed by overlaying of bright field (grey), coral GFP (green) and chlorophyll (red). Chlorophyll fluorescence intensity was quantified using the CellSens software package. For Fig. 4e, a region–of-interest (ROI) was defined as the area bounded by the micropropagate (dashed white line in 4c), and total chlorophyll fluorescence within the ROI quantified for each frame. For Fig. 4f, dynamic ROIs were defined around the two algal cells denoted by blue and red arrows in Fig. 4d. The ROIs were adjusted manually for each frame in Supplementary Movie 4 to track the movements of the algae during the acquisition period. Finally, total chlorophyll fluorescence for each ROI was obtained for each frame.

## Additional information

**How to cite this article:** Shapiro, O. H. *et al*. A coral-on-a-chip microfluidic platform enabling live-imaging microscopy of reef-building corals. *Nat. Commun.* 7:10860 doi: 10.1038/ncomms10860 (2016).

## Supplementary Material

Supplementary Information Supplementary Figures 1-3

Supplementary Movie 1 Polyp bail-out following salinity stress. Time lapse sequence of a small *P. damicornis* branch exposed to salinity stress. Coral fragment was placed in an open Petri dish filled with FASW. Water salinity gradually increased due to evaporation, inducing a polyp bail-out response and the production of multiple clonal micropropagates. Images were captured at 3 min intervals over an 11.5 h period. Scale bar is provided in Fig. 2b

Supplementary Movie 2 Microscopic view of coral calcification. High magnification view of the skeletal growth of a settled *S. pistillata* micropropagate. Here, a Coral on a Chip device holding a 6 days old micropropagate was kept on the microscope stage for a period of 25 h under a constant flow of FASW. Temperature was set to 24^o^C for the entire period. Light flux was maintained at 500 µmol photons m^-2^ s^-1^ for the first 12 h, followed by a 13 h dark period. Skeletal growth was captured during the dark period. Frames were captured at 10 min intervals using differential interference contrast microscopy with a 40X long working distance objective (NA 0.6). Variability in background shading is due to lateral movement of the coral's tissue. Twitching dark circles are shades cast by zooxanthellae above the focal plane. Scale bar is provided in Fig. 3e.

Supplementary Movie 3 Near-complete bleaching of a *P. damicornis* micropropagate following high-light stress. A micropropagate consisting of three polyps was kept on the microscope stage under constant FASW flow, with temperature set to 24^o^C. Light intensity was set to 2500 µmol photons m^-2^ s^-1^ to induce bleaching. Time lapse sequence was captured at 10 min intervals over a period of 8 h using a 4X objective (NA 0.13), with each frame composed of bright field (grey background), coral GFP (green) and algal chlorophyll (red). Scale bar is provided in Fig. 4c.

Supplementary Movie 4 Loss of a single algal symbiont during high-light induced bleaching. A single polyp micropropagate was kept on the microscope stage under constant FASW flow, with temperature set to 24^o^C. Light intensity was set to 1500 µmol photons m^-2^ s^-1^, resulting in a slower bleaching response compared to the experiment shown in Supplementary Movie 3. Time lapse sequence was captured at 5 min intervals over a period of 12 h using a 10X objective (NA 0.3), with each frame composed of bright field (grey background), coral GFP (green) and algal chlorophyll (red). The sequence shows a single algal symbiont (denoted by red arrow in figure 4d) disappearing following an increase in chlorophyll fluorescence. Grey, snake like shadows in the background are the polyp's mesenterial filaments. Scale bar is given in Figure 4d. Frame-by-frame quantification of chlorophyll fluorescence from the disappearing algae and one neighboring algae is given in figure 4f.
