# Corrigendum: A coral-on-a-chip microfluidic platform enabling live-imaging microscopy of reef-building corals

**DOI:** 10.1038/ncomms11680

**Published:** 2016-05-11

**Authors:** Orr H. Shapiro, Esti Kramarsky-Winter, Assaf R. Gavish, Roman Stocker, Assaf Vardi

Nature Communications 7: Article number: 10860 10.1038/ncomms10860 (2016); Published: 03042015; Updated: 05112016

Previous work by Venn *et al*. describing live imaging of coral mounted on cover slips in miniaturized flow cells and live imaging of coral calcification was inadvertently omitted from the reference list of this Article. This work should have been cited in the second paragraph of the introduction, where previous work describing microscopic examination of coral and coral culture systems are referred to, as follows: ‘Previous work by Venn *et al*. described live imaging of coral calcification using the lateral skeleton preparation in combination with miniaturized flow chambers (Venn *et al*. 2011)'. A reference to this work should also have been provided in the section discussing the use of calcein for *in vivo* imaging of coral calcification.

Venn, A., Tambutté, E., Holcomb, M., Allemand, D. & Tambutté, S. Live tissue imaging shows reef corals elevate pH under their calcifying tissue relative to seawater. *PLoS ONE* **6**, e20013 (2011).

