# Supplementary material for: A coral-on-a-chip microfluidic platform enabling live-imaging microscopy of reef-building corals
Source: Nat Commun. 2016 Mar 4;7:10860. doi: 10.1038/ncomms10860 (PMC4785229; doi:10.1038/ncomms10860)
Supplement: Supplementary Information — Supplementary Figures 1-3 [file ncomms10860-s1.pdf]

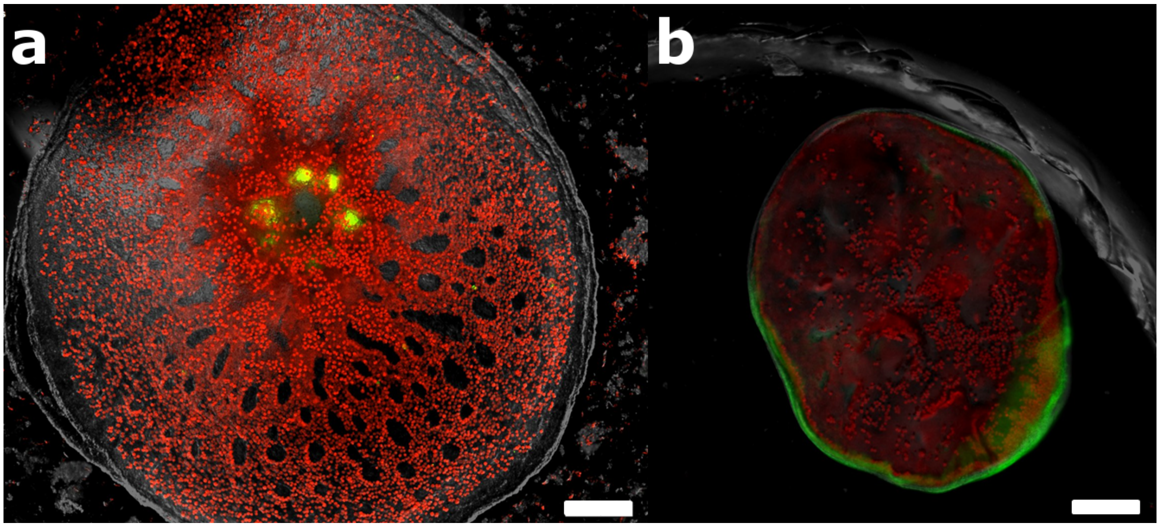

Supplementary Figure 1: Settled micropropagates from additional coral species. Polyps released from the scleractinian corals *Stylophora pistillata* (**a**) and *Seriatopora hystrix* (**b**) via polyp bail-out as described and settled inside microfluidic channels. Each image is composed of three frames captured in sequence. Tissue outline and skeletal features, as well as surrounding biofilm (**a**) and the edge of the 3 mm microwell (**b**), were imaged by dark field microscopy (grey scale). Epifluorescence microscopy was used to capture the auto fluorescence of algal symbionts (red) and the coral's native GFP (green). Scale bars – 200 μm.

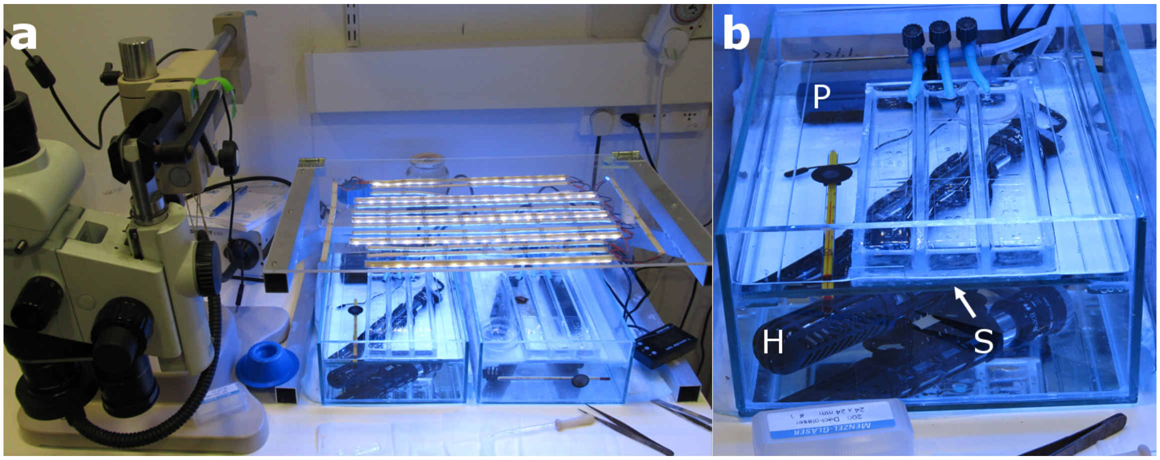

Supplementary Figure 2: Raceway system used for micropropagates settlement and incubation. (**a**) Wide view of the raceway setup, here with two systems are placed side by side. Light is provided by blue and white LED strips glued to a Plexiglas shelf mounted on an aluminum frame. Hinges at the back of the frame allow lifting the shelf to allow easy access to the flow channels as well the use of an overhanging stereoscope (left) for observing micropropagates and tracking settlement progress. (**b**) Close up view of the raceway flow system. The water reservoir, is covered by a glass shelf (S). The raceway itself is placed on top of the glass shelf, with a submersible pump (P) providing continuous flow to the 3 channels. Water overflow from the raceway is returned to the reservoir

through a gap between the glass shelf and container wall. A self-regulated 25W heater (H) controls water temperature.

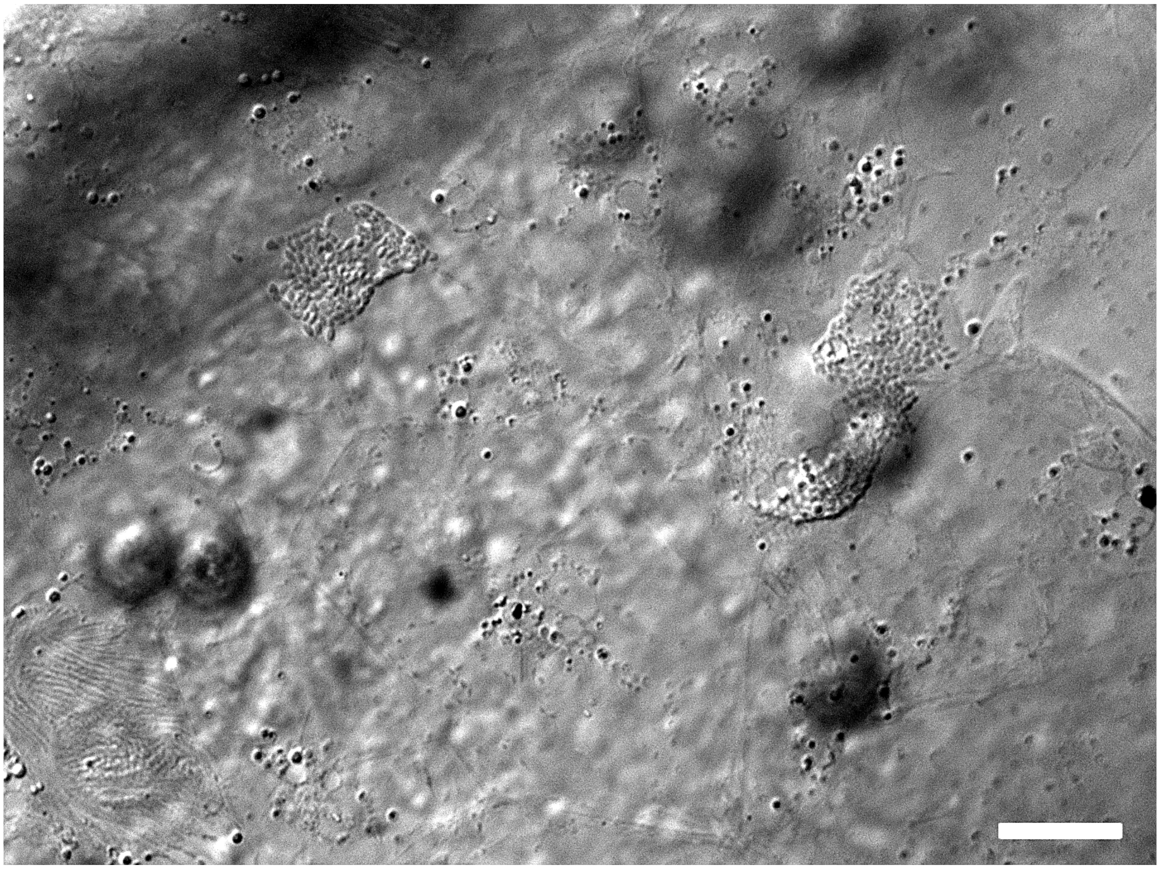

Supplementary Figure 3: High magnification view of the calicoblastic tissue layer of a *P. damicornis* micropropagate. This is an expanded view of figure 2h, demonstrating the level of resolution achievable using the Coral on a Chip microfluidic platform. Three different cell types are clearly discernible, from two cell layers of the calicoblastic tissues: i) Transparent coral host's epidermal cells, squamous and polygonal in shape, are clearly visible, including one with desmoidal processes (bottom left corner); ii) Algal symbionts are here seen slightly blurred as they are located in the gastrodermal cell layer slightly above the focal plane; and iii) Bacterial microcolonies, apparently located between the glass substrate and the calicodermis . It should be noted that this level of resolution requires settling of micropropagates onto a glass coverslip and the use of a high magnification objective with high numerical aperature (here 60X, NA 1.42). Scale bar 20 μm.
